# Supplementary material for: Glial cells undergo rapid changes following acute chemogenetic manipulation of cortical layer 5 projection neurons
Source: Commun Biol. 2024 Oct 9;7:1286. doi: 10.1038/s42003-024-06994-w (PMC11464517; doi:10.1038/s42003-024-06994-w)
Supplement: Supplementary file 2 — Supplementary Information [file 42003_2024_6994_MOESM2_ESM.pdf]

Supplementary Tables

Table 1: Primary and secondary antibodies

**Primary Antibodies**			
**Target**	**Host-Species**	**Concentration**	**Manufacturer**
Anti-cFos	Rabbit	1:500	Synaptic Systems, 226-003
Anti-Iba1	Rabbit	1:500	FUJIFILM Wako 019-19741
Anti-CD68	Mouse	1:500	Abcam, ab955
Anti-vGlut1	Guinea Pig	1:500	Merck Millipore AB5905
Anti-PSD95	Mouse	1:500	Thermo Xe3-1B8
Anti-GFAP	Rabbit	1:500	Dako Z0334
Anti-S100β	Mouse	1:500	Sigma-Aldrich, S2532
Anti-PV	Rabbit	1:500	Swant, PV27
Biotinylated VVA		2 ug/ml	Vector laboratories, B-1235-2
**Secondary Antibodies**			
**Fluorophore**	**Species**	**Concentration**	**Manufacturer**
Alexa Fluor®488	Goat Anti-Rabbit IgG (H+L)	1:500	ThermoFisher, A11034
Alexa Fluor®488	Goat Anti-Mouse IgG (H+L)	1:500	Lifetech, A21041
Alexa Fluor®633	Goat Anti-Guinea Pig IgG (H+L)	1:500	Molecular Probes, A21105
Alexa Fluor®488	Donkey Anti-Rabbit IgG (H+L)	1:500	Invitrogen, A21206
Cy5 streptavidin-conjugated		1:200	Invitrogen, SA1011

Table 2: QuPath cell detection parameters for interneuron subpopulations.

QuPath Parameter	Interneuron Subpopulation	
	PV(+)/VVA(All/+/-)	VVA(+)/PV(All)
Requested Pixel Size (µm)	0.5	0.5
Background Radius (µm)	8.6	6.0
Median Radius (µm)	2.3	3.0
Sigma (µm)	1.5	3.0
Minimum Area (µm)	25.0	60.0
Maximum Area (µm)	400.0	600.0
Detection Threshold	5000.0	200.0
Split by Shape	Yes	Yes
Cell Expansion (µm)	4.0	2.0
Include Nuclei	No	No
Smooth Boundaries	Yes	Yes
Make Measurements	Yes	Yes
Positive Detection Threshold 1	800.0	N/A
Positive Detection Threshold 2	800.0	N/A
Positive Detection Threshold 3	800.0	N/A
Single Threshold	Yes	N/A

Table 3: QuPath object classifiers applied for each of four interneuron subpopulations.

Interneuron Subpopulation		Object Classification	Threshold
PV	VVA		
+	All	PV Area	< 200.0
		PV Circularity	> 0.8
+	+	PV Area	< 200.0
		PV Circularity	> 0.8
		VVA signal intensity standard deviation	> 200.0
+	-	PV Area	< 200.0
		PV Circularity	> 0.8
		VVA signal intensity standard deviation	< 200.0
All	+	VVA signal intensity standard deviation	> 354.9

Table 4: Animal information

Two mice died within 90 min of CNO administration, and before perfusion could be initiated. The brains of these mice were collected but were not included in further data analysis. All animals used for this part of experiments were adults and littermates.

**Rbp4^{Cre}-hM3Dq**					
**Animal ID**	**Sex**	**Genotype**	**Drug**	**Dose**	**Comments and Behaviour**
RCDB2.2A	Male	Rbp4^{Cre}-hM3Dq	CNO	10mg/kg	Animal died, was not used for analysis
RCDB2.2B	Male	Rbp4^{Cre}-hM3Dq	Saline	0.9% NaCl	No changes in behaviour
RCDB2.2C	Male	Rbp4^{Cre}-hM3Dq	CNO	10mg/kg	Sedation-like state
RCDB2.2D	Male	Rbp4^{Cre}-hM3Dq	Saline	0.9% NaCl	No changes in behaviour
RCDB2.2E	Female	Rbp4^{Cre}-hM3Dq	Saline	0.9% NaCl	No changes in behaviour
RCDB2.2F	Female	Rbp4^{Cre}-hM3Dq	CNO	10mg/kg	Sedation-like state
RCDB2.2G	Female	Rbp4^{Cre}-hM3Dq	CNO	10mg/kg	Sedation-like state
RCDB2.2H	Female	Rbp4^{Cre}-hM3Dq	Saline	0.9% NaCl	No changes in behaviour
RCDB3.2B	Male	Rbp4^{Cre}-hM3Dq	CNO	0.5mg/kg	Seemed less active but were easily arousable
RCDB3.2D	Male	Rbp4^{Cre}-hM3Dq	CNO	0.1mg/kg	
RCDB3.2E	Male	Rbp4^{Cre}-hM3Dq	Saline	0.9% NaCl	No changes in behaviour
RCDB3.1A	Male	Rbp4^{Cre}-hM3Dq	CNO	1mg/kg	Animal died, was not used for analysis
RCDB3.1C	Male	Rbp4^{Cre}-hM3Dq	CNO	0.05mg/kg	Mild behavioural effect of reduced activity and slower locomotor activity with no signs of distress
RCDB3.1D	Male	Rbp4^{Cre}-hM3Dq	CNO	0.05mg/kg	
RCDB3.1E	Female	Rbp4^{Cre}-hM3Dq	CNO	0.1mg/kg	Seemed less active but were easily arousable
RCDB3.1F	Female	Rbp4^{Cre}-hM3Dq	Saline	0.9% NaCl	No changes in behaviour
**Rbp4^{Cre}-hM4Di**					
RCDA9.2A	Male	Rbp4^{Cre}-hM4Di	CNO	5mg/kg	No changes in behaviour or welfare were observed
RCDA9.2B	Male	Rbp4^{Cre}-hM4Di	CNO	1mg/kg	
RCDA9.2C	Male	Rbp4^{Cre}-hM4Di	Saline	0.9% NaCl	

RCDA9.2D	Female	Rbp4^{Cre}-hM4Di	CNO	5mg/kg	
RCDA9.2E	Female	Rbp4^{Cre}-hM4Di	CNO	1mg/kg	
RCDA9.2F	Female	Rbp4^{Cre}-hM4Di	Saline	0.9% NaCl	
**Control animals**					
TNPW12.1e	Female	hM3Dq	Saline	0.9% NaCl	No changes in behaviour or welfare were observed
TNPW12.1f	Female	hM3Dq	Saline	0.9% NaCl	
SABX11.1a	Male	hM4Di	Saline	0.9% NaCl	
SABX11.1b	Male	hM4Di	Saline	0.9% NaCl	
RSAB12.2c	Male	Rbp4^{Cre}	Saline	0.9% NaCl	
RSAB12.2f	Female	Rbp4^{Cre}	Saline	0.9% NaCl	
TGJP43.1a	Male	Rbp4^{Cre}	CNO	10mg/kg	
TGJP43.1b	Male	Rbp4^{Cre}	CNO	10mg/kg	
TGJP43.1c	Female	Rbp4^{Cre}	CNO	10mg/kg	
TGJP43.1d	Male	Rbp4^{Cre}	Saline	0.9% NaCl	
TGJP43.1e	Male	Rbp4^{Cre}	Saline	0.9% NaCl	
TGJP43.1f	Male	Rbp4^{Cre}	CNO	10mg/kg	
TGJP43.1g	Female	Rbp4^{Cre}	CNO	10mg/kg	
All animals were perfused 90 min after saline or CNO administration					

Supplementary figures

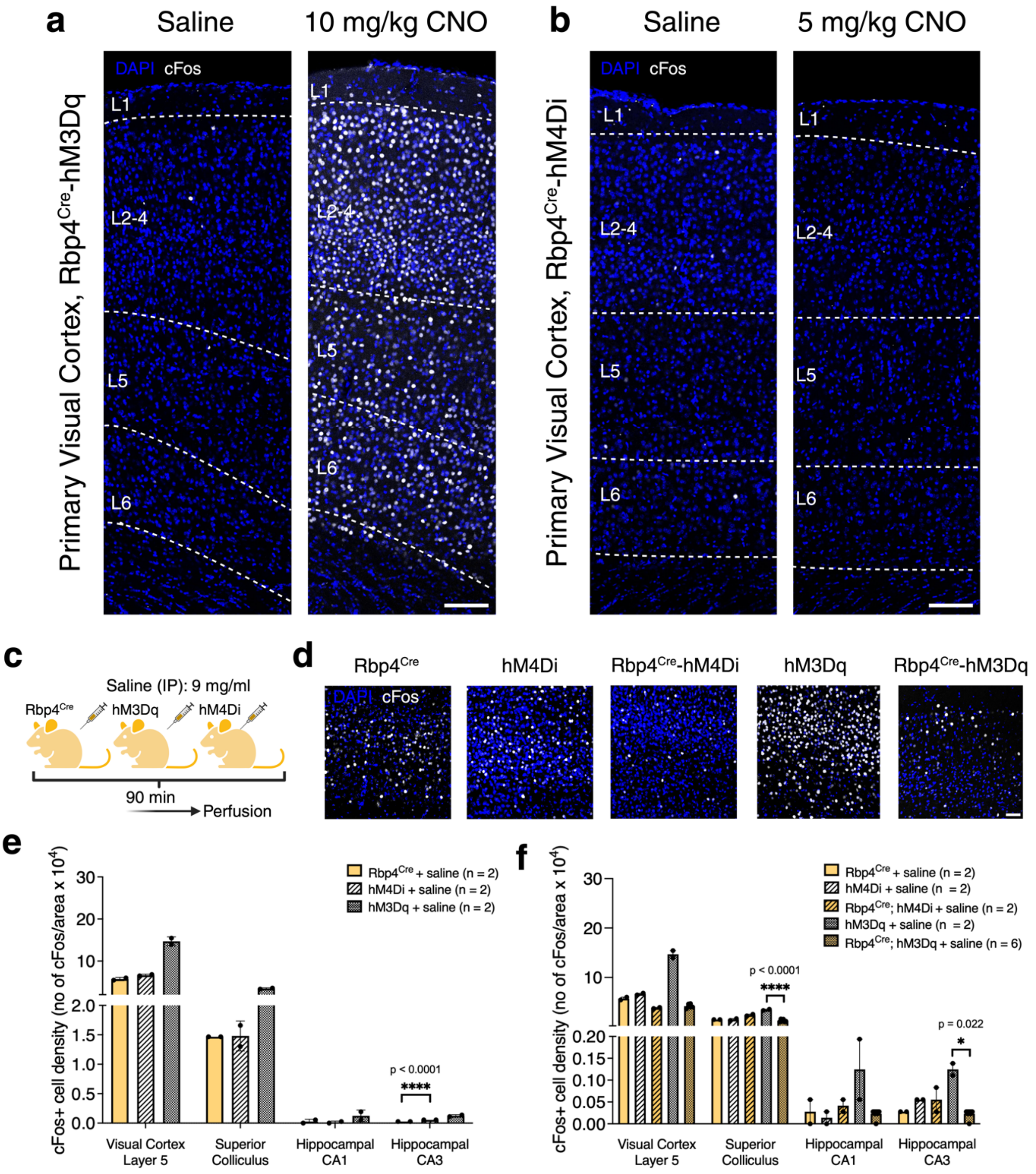

Supplementary Fig. 1: Control experiments

a,b, Representative images of cFos laminar distribution in primary visual cortex in Rbp4^{Cre}-hM3Dq and Rbp4^{Cre}-hM4Di in mice injected with saline and CNO. **c,d,** Cortical layer 5 driver line (Rbp4^{Cre}), inhibitory DREADD receptor line (hM4Di) and excitatory DREADD receptor (hM3Dq) mice were injected only with saline and for validation using cFos immunolabeling. **e,** In visual cortex layer 5, cFos+ density was increased in hM4Di and hM4Dq compared with Rbp4^{Cre}. In SC, increased cFos+ density was higher in hM3Dq than in hM4Di and Rbp4^{Cre}. ***n*** = 2 mouse, with an average of 3 sections per region, evaluated using mixed-effects ANOVA via the corrected method of Benjamini and Yekutieli. **f,** Furthermore, we compared control experiments results with both Rbp4^{Cre}-hM4Di and Rbp4^{Cre}-hM3Dq saline injected mice. In both Rbp4^{Cre}-hM4Di and Rbp4^{Cre}-hM3Dq, cFos+ cell density was lower than hM4Di, hM3Dq and Rbp4^{Cre}, and no differences between transgenic lines were observed. Although, it is important to notice that the

excitatory DREADD receptor line itself has much higher cFos+ density than others suggesting that excitatory DREADD itself has an increased activity without CNO. ***n*** = 2 mouse, with an average of 3 sections per region, evaluated using mixed-effects ANOVA via the corrected method of Benjamini and Yekutieli. All data presented as mean ± SEM, false discovery rate of 0.05 adjusted using Benjamini and Yekutieli, * $p < 0.05$, ** $p < 0.01$, *** $p < 0.001$, **** $p < 0.0001$. Scale bar of **a, b, d** - 100 µm. Detailed statistical information is listed in Supplementary Data 1. Source data is provided as a Supplementary Data 2 file. Created with BioRender.com

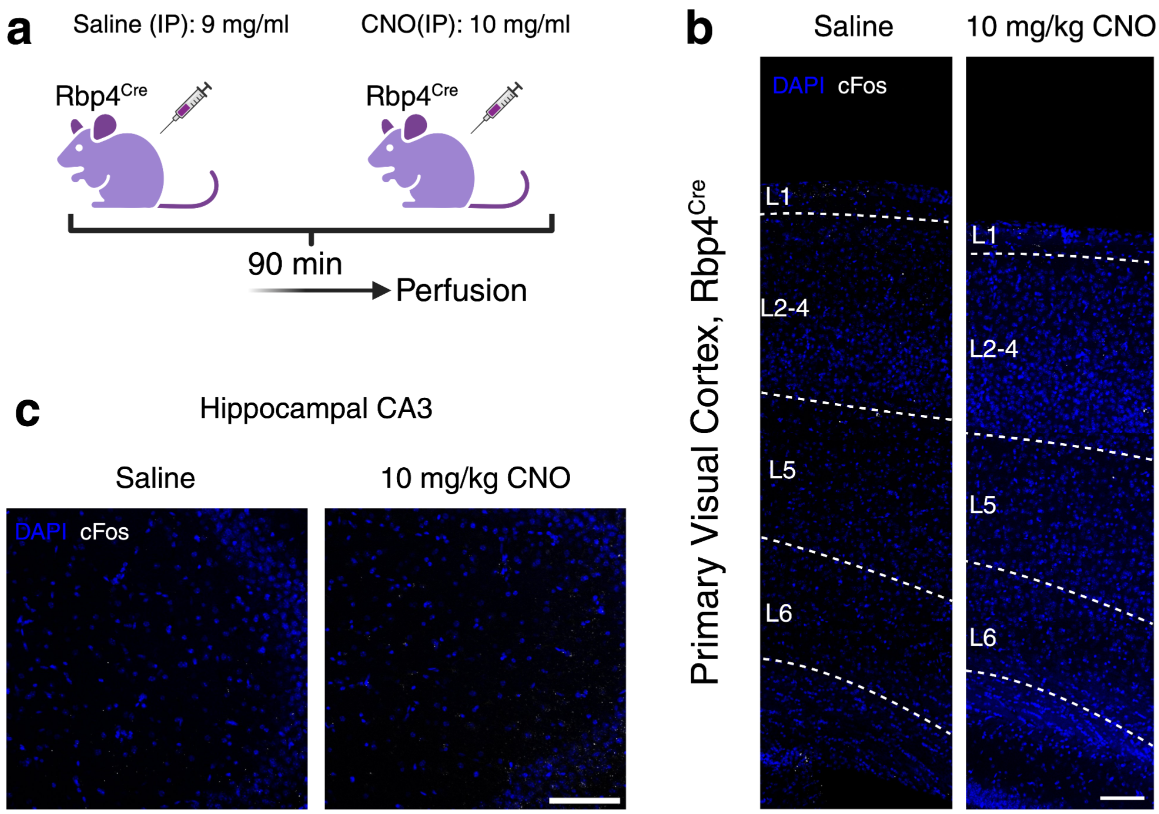

Supplementary Fig. 2: cFos expression in Rbp4^{Cre} mice

a, Schematics of experimental procedure using cortical layer 5 driver line (Rbp4^{Cre}) mice. Rbp4^{Cre} mice were injected with saline and 10 mg/kg CNO and perfused after 90 min. **b,c,** Representative images of cFos immunolabelling in primary visual cortex and hippocampal CA3 regions. Scale bar of **a,b,d** - 100 µm.

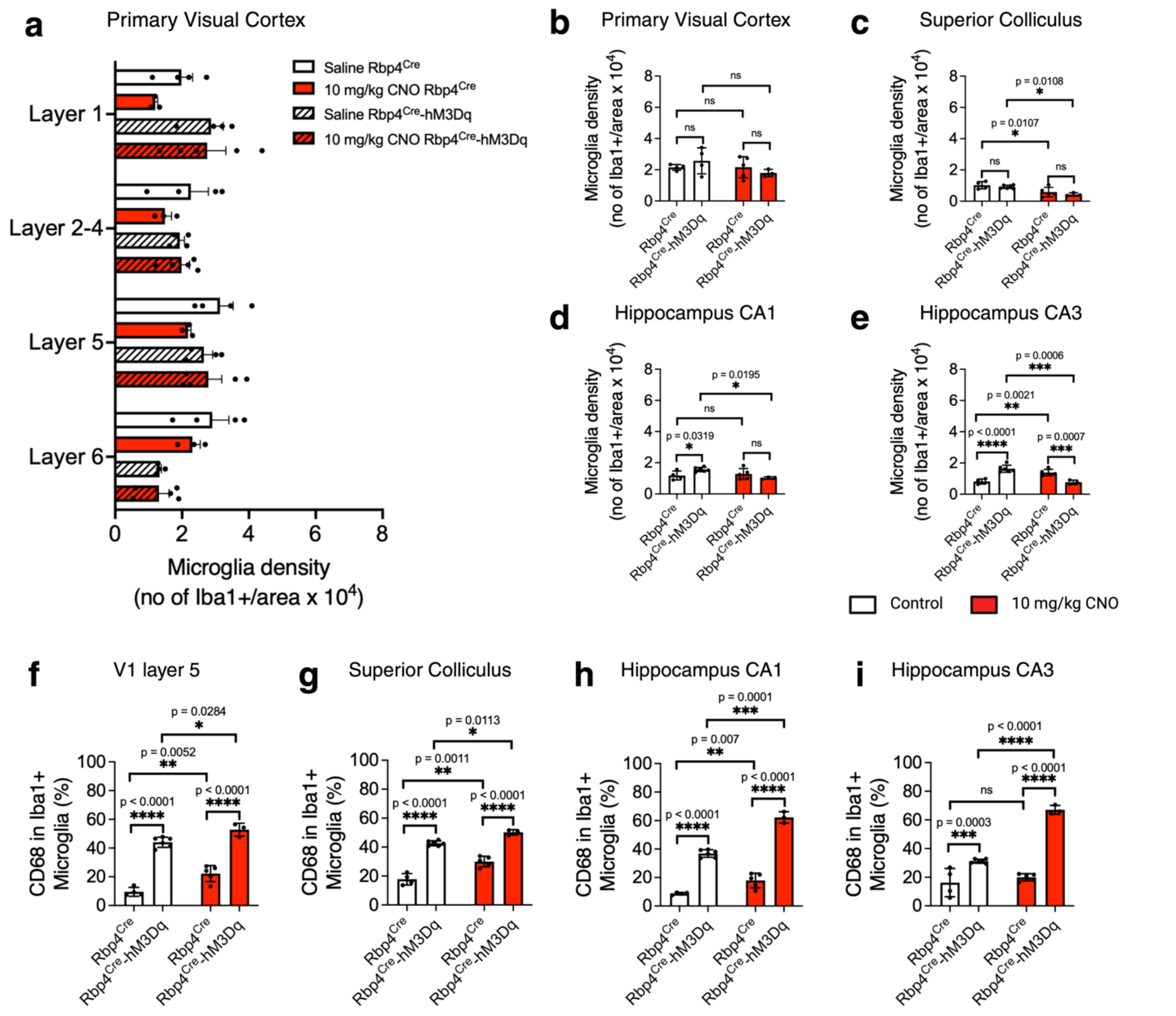

Supplementary Fig. 3: Microglial response to CNO application in Rbp4^{Cre} and Rbp4^{Cre}-hM3Dq mice

a, b, No significant differences in microglial density in primary visual cortex in Rbp4^{Cre} and Rbp4^{Cre}-hM3Dq mice after 10 mg/kg CNO or saline injection. Mice: ***n*** = 4 saline Rbp4^{Cre}, ***n*** = 5 CNO 10 mg/kg Rbp4^{Cre}, ***n*** = 4 saline Rbp4^{Cre}-hM3Dq, ***n*** = 3 CNO 10 mg/kg Rbp4^{Cre}-hM3Dq, with an average of 3 sections per region, a - evaluated using mixed-effects ANOVA via the corrected method of Benjamini and Yekutieli and b - evaluated using two-way ANOVA via the corrected method of Benjamini and Yekutieli. **c**, No significant differences in microglial density in superior colliculus when comparing control Rbp4^{Cre} and Rbp4^{Cre}-hM3Dq mice injected with saline or injected with 10 mg/kg CNO. However, within genotype, microglial density decreased in Rbp4^{Cre} mice after 10 mg/kg CNO injection compared to saline. Similarly, microglial density decreased in Rbp4^{Cre}-hM3Dq mice after 10 mg/kg CNO injection compared with saline. Mice: ***n*** = 4 saline Rbp4^{Cre}, ***n*** = 5 CNO 10 mg/kg Rbp4^{Cre}, ***n*** = 6 saline Rbp4^{Cre}-hM3Dq, ***n*** = 3 CNO 10 mg/kg Rbp4^{Cre}-hM3Dq, with an average of 3 sections per region, evaluated using two-way ANOVA via the corrected method of Benjamini and Yekutieli. **d,** Microglial density is significantly different between control Rbp4^{Cre} and Rbp4^{Cre}-hM3Dq mice injected with saline in CA1 region, but no significant difference between genotypes injected with 10 mg/kg CNO. Microglial density was significantly decreased in Rbp4^{Cre}-hM3Dq mice after 10 mg/kg CNO application compared with saline. Mice: ***n*** = 4 saline Rbp4^{Cre}, ***n*** = 5 CNO 10 mg/kg Rbp4^{Cre}, ***n*** = 6 saline Rbp4^{Cre}-hM3Dq, ***n*** = 3 CNO 10 mg/kg Rbp4^{Cre}-hM3Dq, with an average of 3 sections per region, evaluated using two-way ANOVA via the corrected method of Benjamini and Yekutieli. **e,** Microglial density in CA3 is significantly different in control Rbp4^{Cre} mice and Rbp4^{Cre}-hM3Dq mice injected with saline, and also when comparing Rbp4^{Cre} and Rbp4^{Cre}-hM3Dq mice injected with 10 mg/kg CNO. Microglial density increased in Rbp4^{Cre} mice after 10 mg/kg CNO application compared with saline

but decreased in Rbp4^{Cre}-hM3Dq after CNO application compared with saline. Mice: ***n*** = 4 saline Rbp4^{Cre}, ***n*** = 5 CNO 10 mg/kg Rbp4^{Cre}, ***n*** = 6 saline Rbp4^{Cre}-hM3Dq, ***n*** = 3 CNO 10 mg/kg Rbp4^{Cre}-hM3Dq, with an average of 3 sections per region, evaluated using two-way ANOVA via the corrected method of Benjamini and Yekutieli. **f, g, h, i,** Significant baseline difference in % of Iba1+ microglia that are also CD68+ in primary visual cortex layer 5, superior colliculus, CA1 and CA3 regions between Rbp4^{Cre} and Rbp4^{Cre}-hM3Dq mice injected with saline, and Rbp4^{Cre} and Rbp4^{Cre}-hM3Dq injected with 10 mg/kg CNO. Moreover, injection with 10 mg/kg CNO increased the percentage of Iba1+ microglia that were also CD68+ in all regions tested in both genotypes (Rbp4^{Cre} and Rbp4^{Cre}-hM3Dq). Mice: ***n*** = 4 saline Rbp4^{Cre}, ***n*** = 5 CNO 10 mg/kg Rbp4^{Cre}, ***n*** = 6 saline Rbp4^{Cre}-hM3Dq, ***n*** = 3 CNO 10 mg/kg Rbp4^{Cre}-hM3Dq, with an average of 3 sections per region, evaluated using two-way ANOVA via the corrected method of Benjamini and Yekutieli. All data are presented as mean ± SEM, false discovery rate of 0.05 adjusted using Benjamini and Yekutieli, * $p < 0.05$, ** $p < 0.01$, *** $p < 0.001$, **** $p < 0.0001$. Detailed statistical information is listed in Supplementary Data 1. Source data is provided as a Supplementary Data 2 file. Created with BioRender.com

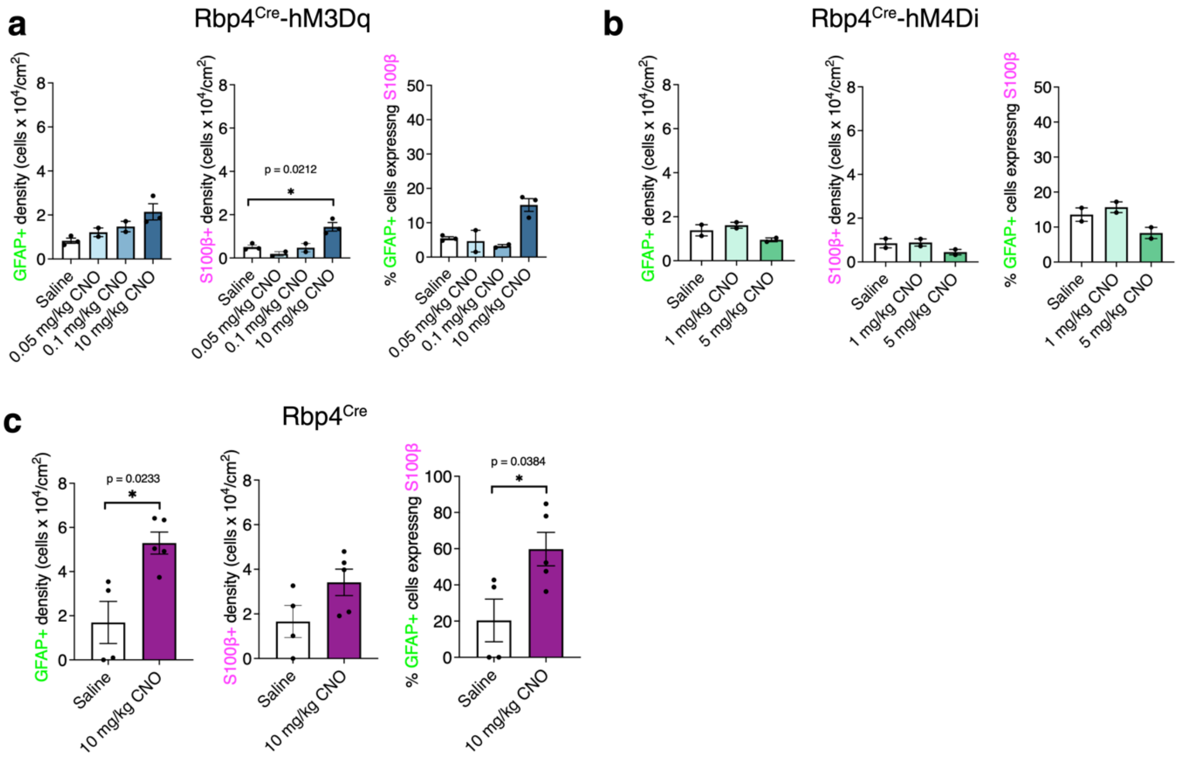

Supplementary Fig. 4: Changes in astrocytes response to CNO application in Rbp4^{Cre}-hM3Dq, Rbp4^{Cre}-hM4Di and Rbp4^{Cre} mice

a, Changes in GFAP+ density, S100β+ density and the proportion of GFAP+ cells expressing S100β after CNO application in Rbp4^{Cre}-hM3Dq mice primary visual cortex. No changes in GFAP+ density was observed. Increased S100β+ density and the proportion of GFAP+ cells expressing S100β were observed with 10 mg/kg CNO. Mice: ***n*** = 4 saline, ***n*** = 2 CNO 0.05 mg/kg, ***n*** = 2 CNO 0.1 mg/kg, ***n*** = 3 CNO 10 mg/kg, with an average of 3 sections per region, evaluated using mixed-effects ANOVA via the corrected method of Benjamini and Yekutieli. **b,** No significant changes in GFAP+ density, S100β+ density and the proportion of GFAP+ cells expressing S100β after CNO application in Rbp4^{Cre}-hM4Di mice primary visual cortex. Mice: ***n*** = 2 saline, ***n*** = 2 CNO 1 mg/kg, ***n*** = 2 CNO 5 mg/kg, with an average of 3 sections per region, evaluated using mixed-effects ANOVA via the corrected method of Benjamini and Yekutieli. **c,** Changes in GFAP+ density, S100β+ density and the proportion of GFAP+ cells expressing S100β after CNO application in Rbp4^{Cre} mice primary visual cortex. Increased GFAP+ density and the proportion of GFAP+ cells expressing S100β were observed with 10 mg/kg CNO. No changes in S100β+ density was observed with 10 mg/kg CNO. Mice: ***n*** = 4 saline, ***n*** = 5 CNO 10 mg/kg, with an average of 3 sections per

region and per mouse, evaluated using mixed-effects ANOVA via Holm-Šidak's multiple comparisons test. All data are presented as mean ± SEM, false discovery rate of 0.05 adjusted using Benjamini and Yekutieli, * $p < 0.05$, ** $p < 0.01$, *** $p < 0.001$, **** $p < 0.0001$. Detailed statistical information is listed in Supplementary Data 1. Source data is provided as a Supplementary Data 2 file. Created with BioRender.com

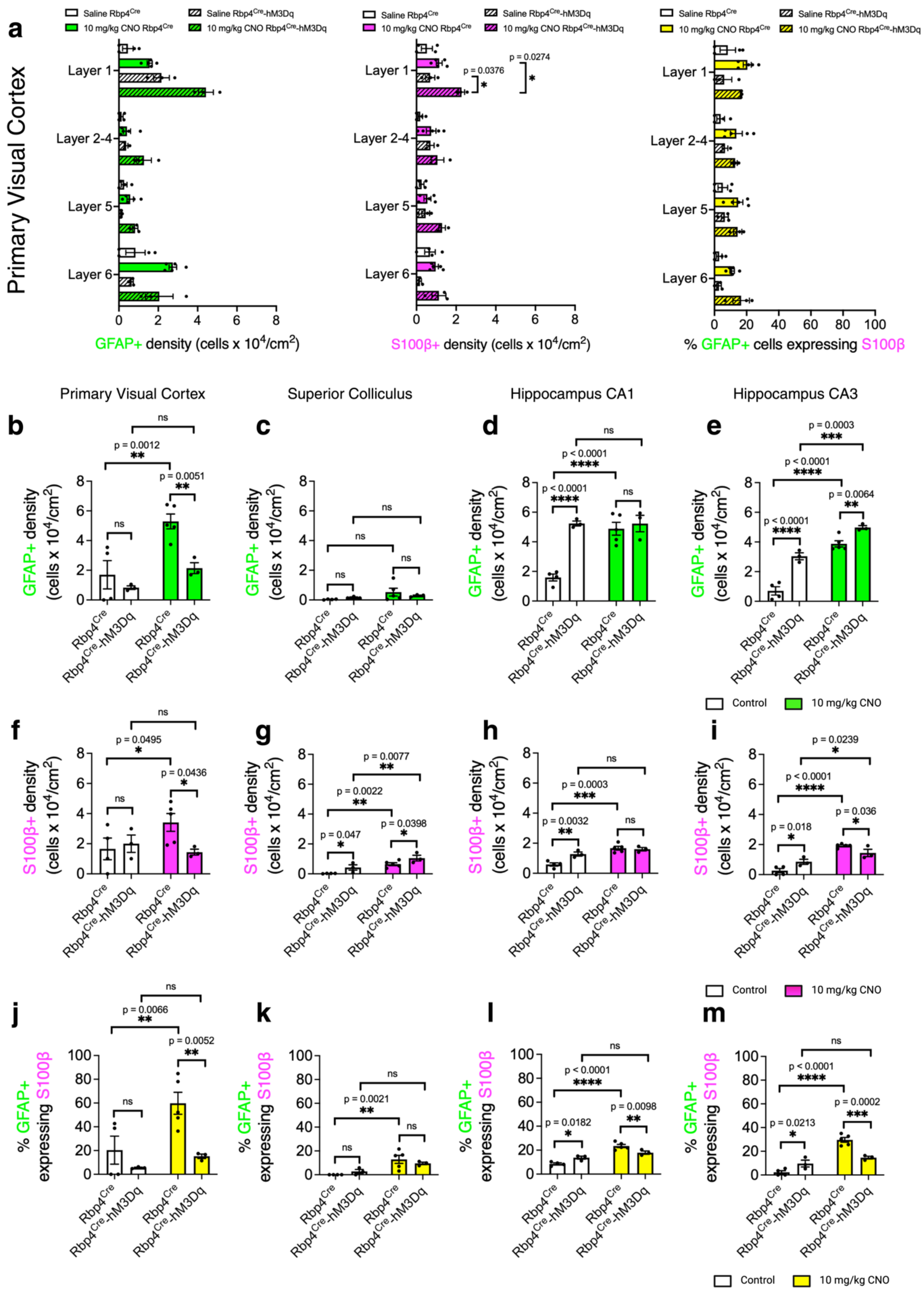

Supplementary Fig. 5: Changes in astrocyte response to CNO application between Rbp4^{Cre} and Rbp4^{Cre}-hM3Dq mice

a, No significant changes in GFAP+ and the proportion of GFAP+ cells expressing S100β in primary visual cortex between Rbp4^{Cre} and Rbp4^{Cre}-hM3Dq mice after 10 mg/kg CNO application. S100β density increased in Rbp4^{Cre}-hM3Dq after 10 mg/kg CNO application compared with Rbp4^{Cre}-hM3Dq saline and

compared to $Rbp4^{Cre}$- mice injected with 10 mg/kg CNO. Mice: ***n*** = 4 saline $Rbp4^{Cre}$, ***n*** = 5 CNO 10 mg/kg $Rbp4^{Cre}$, ***n*** = 4 saline $Rbp4^{Cre}$-hM3Dq, ***n*** = 3 CNO 10 mg/kg $Rbp4^{Cre}$-hM3Dq, with an average of 3 sections per region, evaluated using mixed-effects ANOVA via the corrected method of Benjamini and Yekutieli. **b, c, d, e,** Changes in GFAP+ density in primary visual cortex, superior colliculus, CA1 and CA3 regions between: $Rbp4^{Cre}$ and $Rbp4^{Cre}$-hM3Dq injected with saline; $Rbp4^{Cre}$ and $Rbp4^{Cre}$-hM3Dq injected with 10 mg/kg CNO; $Rbp4^{Cre}$ injected with saline and 10 mg/kg CNO; $Rbp4^{Cre}$-hM3Dq injected with saline and 10 mg/kg CNO. Mice: ***n*** = 4 saline $Rbp4^{Cre}$, ***n*** = 5 CNO 10 mg/kg $Rbp4^{Cre}$, ***n*** = 4 saline $Rbp4^{Cre}$-hM3Dq, ***n*** = 3 CNO 10 mg/kg $Rbp4^{Cre}$-hM3Dq, with an average of 3 sections per region, evaluated using two-way ANOVA via the corrected method of Benjamini and Yekutieli. **f, g, h, i,** Changes in S100β+ density in primary visual cortex, superior colliculus, CA1 and CA3 regions between: $Rbp4^{Cre}$ and $Rbp4^{Cre}$-hM3Dq injected with saline; $Rbp4^{Cre}$ and $Rbp4^{Cre}$-hM3Dq injected with 10 mg/kg CNO; $Rbp4^{Cre}$ injected with saline and 10 mg/kg CNO; $Rbp4^{Cre}$-hM3Dq injected with saline and 10 mg/kg CNO. Mice: ***n*** = 4 saline $Rbp4^{Cre}$, ***n*** = 5 CNO 10 mg/kg $Rbp4^{Cre}$, ***n*** = 4 saline $Rbp4^{Cre}$-hM3Dq, ***n*** = 3 CNO 10 mg/kg $Rbp4^{Cre}$-hM3Dq, with an average of 3 sections per region, evaluated using two-way ANOVA via the corrected method of Benjamini and Yekutieli. **j, k, l, m,** Changes in the proportion of GFAP+ cells expressing S100β in primary visual cortex, superior colliculus, CA1 and CA3 regions between: $Rbp4^{Cre}$ and $Rbp4^{Cre}$-hM3Dq injected with saline; $Rbp4^{Cre}$ and $Rbp4^{Cre}$-hM3Dq injected with 10 mg/kg CNO; $Rbp4^{Cre}$ injected with saline and 10 mg/kg CNO; $Rbp4^{Cre}$-hM3Dq injected with saline and 10 mg/kg CNO. Mice: ***n*** = 4 saline $Rbp4^{Cre}$, ***n*** = 5 CNO 10 mg/kg $Rbp4^{Cre}$, ***n*** = 4 saline $Rbp4^{Cre}$-hM3Dq, ***n*** = 3 CNO 10 mg/kg $Rbp4^{Cre}$-hM3Dq, with an average of 3 sections per region, evaluated using two-way ANOVA via the corrected method of Benjamini and Yekutieli. All data are presented as mean ± SEM, false discovery rate of 0.05 adjusted using Benjamini and Yekutieli, * $p < 0.05$, ** $p < 0.01$, *** $p < 0.001$, **** $p < 0.0001$. Detailed statistical information is listed in Supplementary Data 1. Source data is provided as a Supplementary Data 2 file. Created with BioRender.com

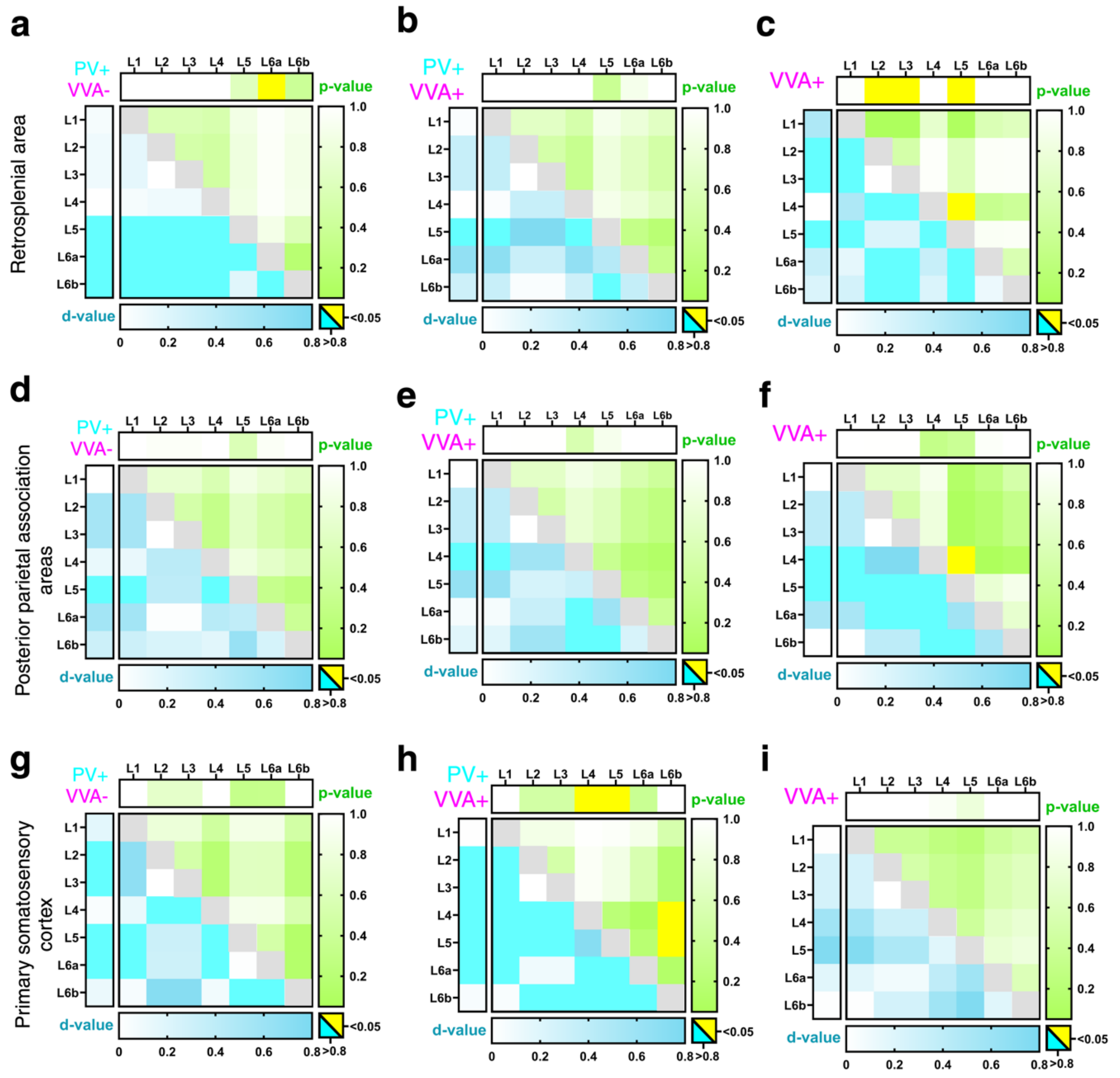

Supplementary Fig. 6: Changes in PV and VVA in different cortical regions in Rbp4^{Cre}-hM3Dq mice

a, b, c, Retrosplenial area. **d, e, f,** Posterior parietal association areas. **g, h, i,** Primary somatosensory cortex. Mice: ***n*** = 3 saline, ***n*** = 3 CNO 10 mg/kg. All heat maps illustrate the effects of 10 mg/kg CNO administration within and between brain regions on PV+/VVA-, PV+/VVA+ and VVA+ interneuron densities. Shades of blue indicate effect size as Cohen's d - values (cyan for large effect sizes > 0.8), and shades of green indicate statistical significance as p - values (yellow for p < 0.05). Horizontal bars indicate the overall within-region/layer statistical significance of CNO administration evaluated using two-way ANOVA (Šidak's multiple comparisons), and vertical bars indicate the overall within-region/layer effect size of CNO administration evaluated using Cohen's d. Pairwise comparisons between cortical layers/regions are represented in the body of each heat map, with statistical significance calculated by applying the student's t-test to outcomes of the two-stage step-up method of Benjamini, Krieger and Yekutieli and effect size calculated via Cohen's d. Detailed statistical information is listed in Supplementary Data 1. Source data is provided as a Supplementary Data 2 file. Created with BioRender.com

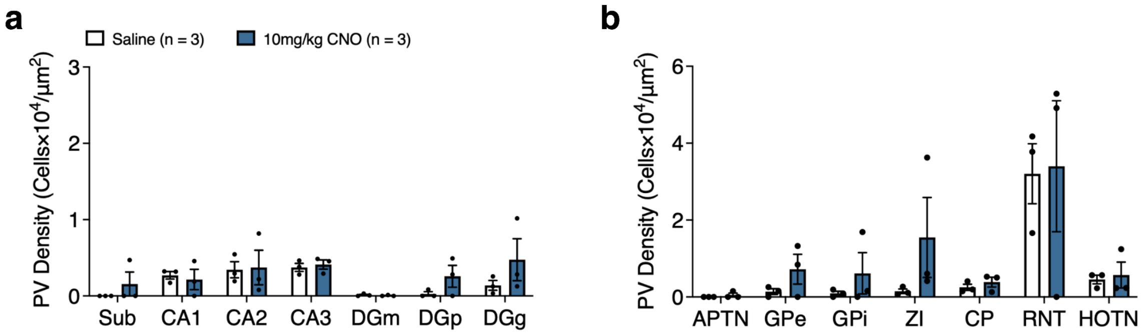

Supplementary Fig. 7: PV+ interneuron density in Rbp4^{Cre}-hM3Dq mice hippocampus and subcortical regions

a, b, Changes in PV+ cell density in hippocampus and other subcortical regions after 10 mg/kg CNO application. Mice: ***n*** = 3 saline, ***n*** = 3 CNO 10 mg/kg, average of 3 sections per region and per mouse, using mixed-effects ANOVA via the corrected method of Benjamini and Yekutieli. All data are presented as mean ± SEM, false discovery rate of 0.05 adjusted using Benjamini and Yekutieli, * $p < 0.05$, ** $p < 0.01$, *** $p < 0.001$, **** $p < 0.0001$. Sub - subiculum; CA1, CA2, CA3 - cornu ammonis 1, 2, 3; DGm - dentate gyrus molecular layer; DGp - dentate gyrus polymorph layer; DGg - dentate gyrus granule layer; APTN - anterior pretectal nucleus; GPe - globus pallidus external segment; GPi - globus pallidus internal segment; ZI - zona incerta; CP - caudoputamen; RNT - reticular nucleus thalamus; HOTN - higher order thalamic nuclei. Detailed statistical information is listed in Supplementary Data 1. Source data is provided as a Supplementary Data 2 file. Created with BioRender.com

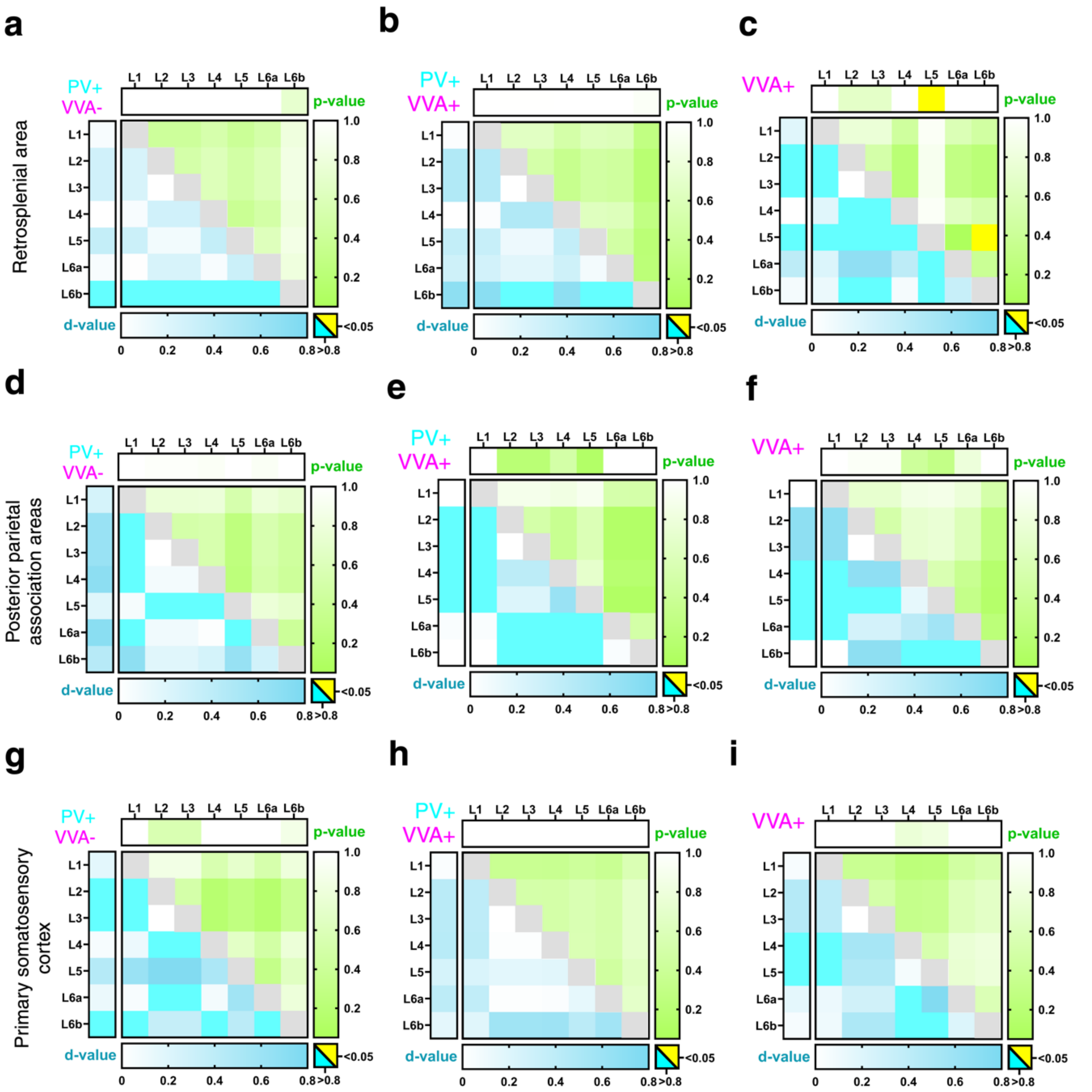

Supplementary Fig. 8: Changes in PV and VVA in different cortical regions in Rbp4^{Cre}-hM4Di mice

a, b, c, Retrosplenial area. **d, e, f,** Posterior parietal association areas. **g, h, i,** Primary somatosensory cortex. Mice: ***n*** = 2 saline, ***n*** = 2 CNO 1 mg/kg. All heat maps illustrate the effects of 1 mg/kg CNO administration within and between brain regions on PV+/VVA-, PV+/VVA+ and VVA+ interneuron densities. Shades of blue indicate effect size as Cohen's d - values (cyan for large effect sizes > 0.8), and shades of green indicate statistical significance as p - values (yellow for p < 0.05). Horizontal bars indicate the overall within-region/layer statistical significance of CNO administration evaluated using two-way ANOVA (Šidak's multiple comparisons), and vertical bars indicate the overall within-region/layer effect size of CNO administration evaluated using Cohen's d. Pairwise comparisons between cortical layers/regions are represented in the body of each heat map, with statistical significance calculated by applying the student's t-test to outcomes of the two-stage step-up method of Benjamini, Krieger and Yekutieli and effect size calculated via Cohen's d. Detailed statistical information is listed in Supplementary Data 1. Source data is provided as a Supplementary Data 2 file. Created with BioRender.com

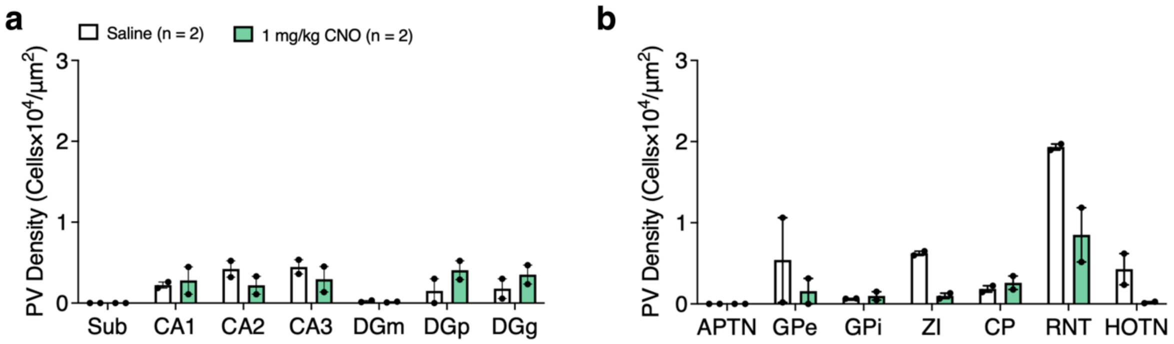

Supplementary Fig. 9: PV+ interneuron density in Rbp4^{Cre}-hM4Di mice hippocampus and subcortical regions

a, b, Changes in PV+ cell density in hippocampus and other subcortical regions after 1 mg/kg CNO application. Mice: ***n*** = 2 saline, ***n*** = 2 CNO 1 mg/kg, average of 3 sections per region and per mouse, using mixed-effects ANOVA via the corrected method of Benjamini and Yekutieli. All data are presented as mean ± SEM, false discovery rate of 0.05 adjusted using Benjamini and Yekutieli, * $p < 0.05$, ** $p < 0.01$, *** $p < 0.001$, **** $p < 0.0001$. Sub - subiculum; CA1, CA2, CA3 - cornu ammonis 1, 2, 3; DGm - dentate gyrus molecular layer; DGp - dentate gyrus polymorph layer; DGg - dentate gyrus granule layer; APTN - anterior pretectal nucleus; GPe - globus pallidus external segment; GPi - globus pallidus internal segment; ZI - zona incerta; CP - caudoputamen; RNT - reticular nucleus thalamus; HOTN - higher order thalamic nuclei. Detailed statistical information is listed in Supplementary Data 1. Source data is provided as a Supplementary Data 2 file. Created with BioRender.com
