# Supplementary material for: Glial cells undergo rapid changes following acute chemogenetic manipulation of cortical layer 5 projection neurons
Source: Commun Biol. 2024 Oct 9;7:1286. doi: 10.1038/s42003-024-06994-w (PMC11464517; doi:10.1038/s42003-024-06994-w)
Supplement: Supplementary file 3 — Description of Additional Supplementary File [file 42003_2024_6994_MOESM3_ESM.pdf]

Description of Additional Supplementary Files

File name: Supplementary Data 1
Description: Supplementary Statistics – we provide all statistical analysis for all main and supplementary figures

File name: Supplementary Data 2
Description: Source data – we provide all raw data (averaged by animal or by cell) for all main and supplementary figures
