# Supplementary material for: Glial cells undergo rapid changes following acute chemogenetic manipulation of cortical layer 5 projection neurons
Source: Commun Biol. 2024 Oct 9;7:1286. doi: 10.1038/s42003-024-06994-w (PMC11464517; doi:10.1038/s42003-024-06994-w)
Supplement: Supplementary file 6 — Reporting Summary [file 42003_2024_6994_MOESM6_ESM.pdf]

nature portfolio

Corresponding author(s): Dr. Auguste Vadisiute (auguste.vadisiute@dpag.ox.ac.uk) Prof. Zoltán Molnár (zoltan.molnar@dpag.ox.ac.uk)

Last updated by author(s): Aug 12, 2024

Reporting Summary

Nature Portfolio wishes to improve the reproducibility of the work that we publish. This form provides structure for consistency and transparency in reporting. For further information on Nature Portfolio policies, see our Editorial Policies and the Editorial Policy Checklist.

Statistics

For all statistical analyses, confirm that the following items are present in the figure legend, table legend, main text, or Methods section.

n/a	Confirmed	
☐	☒	The exact sample size (*n*) for each experimental group/condition, given as a discrete number and unit of measurement
☐	☒	A statement on whether measurements were taken from distinct samples or whether the same sample was measured repeatedly
☐	☒	The statistical test(s) used AND whether they are one- or two-sided<br>*Only common tests should be described solely by name; describe more complex techniques in the Methods section.*
☐	☒	A description of all covariates tested
☐	☒	A description of any assumptions or corrections, such as tests of normality and adjustment for multiple comparisons
☐	☒	A full description of the statistical parameters including central tendency (e.g. means) or other basic estimates (e.g. regression coefficient) AND variation (e.g. standard deviation) or associated estimates of uncertainty (e.g. confidence intervals)
☐	☒	For null hypothesis testing, the test statistic (e.g. *F*, *t*, *r*) with confidence intervals, effect sizes, degrees of freedom and *P* value noted<br>*Give P values as exact values whenever suitable.*
☒	☐	For Bayesian analysis, information on the choice of priors and Markov chain Monte Carlo settings
☒	☐	For hierarchical and complex designs, identification of the appropriate level for tests and full reporting of outcomes
☐	☒	Estimates of effect sizes (e.g. Cohen's *d*, Pearson's *r*), indicating how they were calculated

Our web collection on statistics for biologists contains articles on many of the points above.

Software and code

Policy information about availability of computer code

Data collection	n/a
Data analysis	1. For immunostaining data analysis:<br>1.1. Open source code scikit-image (https://scikit-image.org/docs/stable/api/skimage.morphology.html), skimage.filters (https://scikit-image.org/docs/stable/api/skimage.filters.html), and Scikit-image Python package (https://scikit-image.org);<br>1.2. Jupyter notebook https://jupyter.org and Anaconda https://www.anaconda.com<br>1.3. ImageJ/Fiji 2.9.0 v1.54b https://imagej.nih.gov/ij/download.html;<br>1.4. QuPath (v0.4.2) https://qupath.github.io;<br>1.5. QUINT pipeline https://quint-workflow.readthedocs.io/en/latest/index.html<br>1.6. QuickNII atlas registration software (RRID:SCR_016854)<br>1.7. Allen Mouse Common Coordinate Framework 3D reference atlas (2017 CCFv3)<br>1.8. VisuAlign software for nonlinear refinement (v0.8 RRID:SCR_017978)<br>1.9. Nutil software https://nutil.readthedocs.io/en/latest/<br>2. Electrophysiology<br>2.1. pClamp 10.5<br>3. Statistics<br>3.1. GraphPad Prism 9 https://www.graphpad.com

For manuscripts utilizing custom algorithms or software that are central to the research but not yet described in published literature, software must be made available to editors and reviewers. We strongly encourage code deposition in a community repository (e.g. GitHub). See the Nature Portfolio guidelines for submitting code & software for further information.

Data

Policy information about availability of data

All manuscripts must include a data availability statement. This statement should provide the following information, where applicable:

- Accession codes, unique identifiers, or web links for publicly available datasets
- A description of any restrictions on data availability
- For clinical datasets or third party data, please ensure that the statement adheres to our policy

Statistical analysis is provided as supplementary statistics and raw data is provided as source data. Images and all data will be made available upon request. Custom script (quint_postprocessing_subdivisions.m) has been deposited on GitHub (https://github.com/marhmue/2023_DREADD_Interneurons.git).

Research involving human participants, their data, or biological material

Policy information about studies with human participants or human data. See also policy information about sex, gender (identity/presentation), and sexual orientation and race, ethnicity and racism.

Reporting on sex and gender	n/a
Reporting on race, ethnicity, or other socially relevant groupings	n/a
Population characteristics	n/a
Recruitment	n/a
Ethics oversight	n/a

Note that full information on the approval of the study protocol must also be provided in the manuscript.

Field-specific reporting

Please select the one below that is the best fit for your research. If you are not sure, read the appropriate sections before making your selection.

☒ Life sciences ☐ Behavioural & social sciences ☐ Ecological, evolutionary & environmental sciences

For a reference copy of the document with all sections, see nature.com/documents/nr-reporting-summary-flat.pdf

Life sciences study design

All studies must disclose on these points even when the disclosure is negative.

Sample size	Samples size was calculated using G power and including different treatment conditions. For patch clamp recording excitatory and inhibitory DREADDs sample sizes were 7 for each chemogenetic manipulation. For immunohistohemistry experiments we used 2 mice per dose for inhibitory DREADD and 2 to 4 mice per dose for excitatory DREADD. Sample size (n) refers to individual animals for microglial density, microglial CD68+, GFAP+ and S100β+ cells density, synaptic density, and cFos+ cell density, while it refers to individual microglia for morphological analysis (microglial volume, microglial soma size and the number of primary branches, and 3D Sholl analysis). To evaluate glial cell density, S100β+ cell density and microglial activity in visual cortex layer 5 (n = 3 images/animal), superior colliculus (n = 6 images/animal) and hippocampus CA1 and CA3 regions (n = 3 images/animal). To examine individual microglial cell morphological features and vGlut1/PSD95 synaptic density in visual cortex layer 5 (n = 9 images/animal) and hippocampus CA3 region (n = 9 images/animal). Interneuron densities were computed for each brain with 1-3 technical replicate sections per animal, genotype, and DREADD injection condition (1-2 for Rbp4Cre-hM4Di saline controls; 3 for Rbp4Cre-hM4Di 5 mg/kg CNO administration; 1-3 for Rbp4Cre-hM4Dq saline controls; and 1-2 for Rbp4Cre-hM4Dq 10 mg/kg CNO administration - see the Source Data file for more details).
Data exclusions	From data analysis we exclude all animals with n=1 and dead animals after CNO application.
Replication	For 3D microglia imaging and 3D synaptic density analysis we took 9 images/mouse; for interneurons 1-3 images/mouse and for the rest immunohistochemistry experiments 3 images/mouse. Technical replicate sections per animal, genotype, and DREADD injection condition - see the Source Data file for more details.
Randomization	Allocation for animals were not random. For all experiments, animals were litter-matched.
Blinding	After CNO application, immunohistochemistry, imaging and data analyses were conducted blind to animal genotype, CNO or saline injection. All electrophysiology experiments were performed blind to animal genotype.

Reporting for specific materials, systems and methods

We require information from authors about some types of materials, experimental systems and methods used in many studies. Here, indicate whether each material, system or method listed is relevant to your study. If you are not sure if a list item applies to your research, read the appropriate section before selecting a response.

Materials & experimental systems

n/a	Involved in the study
☐	☒ Antibodies
☒	☐ Eukaryotic cell lines
☒	☐ Palaeontology and archaeology
☐	☒ Animals and other organisms
☒	☐ Clinical data
☒	☐ Dual use research of concern
☒	☐ Plants

Methods

n/a	Involved in the study
☒	☐ ChIP-seq
☒	☐ Flow cytometry
☒	☐ MRI-based neuroimaging

Antibodies

Antibodies used

rabbit anti-cFos (1:500, Synaptic Systems, 226-003), rabbit anti-Iba1 (1:500, FUJIFILM Wako 019-19741), mouse anti-CD68 (1:500, Abcam, ab955), guinea pig anti-vGlut1 (1:500, Merck Millipore AB5905), mouse anti-PSD95 (1:500, ThermoFisher 7E3-1B8), rabbit anti-GFAP (1:500, Dako Z0334), mouse anti-S100β (1:500, Sigma-Aldrich, S2532), rabbit anti-PV (1:500, PV27, Swant), biotinylated VVA (B-1235-2, Vector laboratories), goat anti-rabbit A488 (1:500, ThermoFisher, A11034), goat anti-mouse A488 (1:500, Lifetech, A21041), goat anti-guinea pig A633 (1:500, Molecular Probes, A21105), donkey anti-rabbit A488 (1:500, Invitrogen, A21206) and Cy5 streptavidin-conjugated (1:200, Invitrogen, SA1011).

Validation

The validation of each antibody are listed on the websites of corresponding manufactures:
- rabbit anti-cFos (1:500, Synaptic Systems, 226-003) this antibody has been recently replaced by monoclonal recombinant rabbit antibody. Validated for ICC, IHC and IHC-P (https://sysy.com/product/226003#:~:text=This%20antibody%20has%20been%20replaced,%2C%20IHC%20and%20IHC%2DP.);

- rabbit anti-Iba1 (1:500, FUJIFILM Wako 019-19741) has been validated for use in IHC and ICC as stated on the product paged and has been referenced in at least 8 papers (https://labchem-wako.fujifilm.com/us/product/detail/W01W0101-1974.html#:~:text=FUJIFILM%20Wako%27s%20%22Anti%20Iba1%2C%20Rabbit,a%20microglia%20marker%20antibody%20standard);

- mouse anti-CD68 (1:500, Abcam, ab955) has been validated for use in ICC/IF, WB, IHC. This product has switched from a hybridoma to recombinant production method on 21st September 2020. This antibody had been referenced in at least 527 papers (https://www.abcam.com/products/primary-antibodies/cd68-antibody-kp1-ab955.html);

- guinea pig anti-vGlut1 (1:500, Merck Millipore AB5905) has been validated for use in IHC and has been referenced in at least 10 papers (https://www.merckmillipore.com/GB/en/product/Anti-Vesicular-Glutamate-Transporter-1-Antibody,MM_NF-AB5905#documentation);

- mouse anti-PSD95 (1:500, ThermoFisher 7E3-1B8) has been validated for use in WB, IF, IHC, ICC/IF, Flow, IP and has been referenced in at least 230 papers. This antibody was verified by Knockout to ensure that the antibody binds to the antigen stated. (https://www.thermofisher.com/antibody/product/PSD-95-Antibody-clone-7E3-1B8-Monoclonal/MA1-046);

- rabbit anti-GFAP (1:500, Dako Z0334) has been validated for use in IHC, ICC, IHC-P, IHC-F, and knockout validation and has been referenced in at least 410 papers (https://www.labome.com/product/Dako/Z0334.html);

- mouse anti-S100β (1:500, Sigma-Aldrich, S2532) has been validated for use in IHC, ELISA, IF, WB and has been referenced in at least 290 papers (https://www.sigmaaldrich.com/GB/en/product/sigma/s2532);

- rabbit anti-PV (1:500, PV27, Swant) has been validated for use in IHC and WB and has been referenced in at least 4 papers (https://www.swant.com/pdfs/x_Rabbit_anti_parvalbumin_PV27.pdf);

- biotinylated VVA (B-1235-2, Vector laboratories) has been validated for use in IHC, IHC-F, IHC-P, ICC, IF, ELISPOT and has been referenced in at least 9 papers (https://www.2bscientific.com/Products/Vector-Laboratories/B-1235-2/Vicia-Villosa-Lectin-VVL-VVA-Biotinylated);

- goat anti-rabbit A488 (1:500, ThermoFisher, A11034) has been validated as stated on the product page and has been referenced in 6327 papers (https://www.thermofisher.com/antibody/product/Goat-anti-Rabbit-IgG-H-L-Highly-Cross-Adsorbed-Secondary-Antibody-Polyclonal/A-11034);

- goat anti-mouse A488 (1:500, Lifetech, A21042) has been validated as stated on the product page and has been referenced in 435 papers (https://www.thermofisher.com/antibody/product/Goat-anti-Mouse-IgM-Heavy-chain-Cross-Adsorbed-Secondary-Antibody-Polyclonal/A-21042);

- goat anti-guinea pig A633 (1:500, Molecular Probes, A21105) has been validated as stated on the product page and has been referenced in 87 papers (https://www.thermofisher.com/antibody/product/Goat-anti-Guinea-Pig-IgG-H-L-Highly-Cross-Adsorbed-Secondary-Antibody-Polyclonal/A-21105);

- donkey anti-rabbit A488 (1:500, Invitrogen, A21206) has been validated as stated on the product page and has been referenced in 6178 papers (https://www.thermofisher.com/antibody/product/Donkey-anti-Rabbit-IgG-H-L-Highly-Cross-Adsorbed-Secondary-Antibody-Polyclonal/A-21206);

- Cy5 streptavidin-conjugated (1:200, Invitrogen, SA1011) has been validated as stated on the product page (https://www.fishersci.com/shop/products/streptavidin-cy5-1/501121551)

Animals and other research organisms

Policy information about studies involving animals; ARRIVE guidelines recommended for reporting animal research, and Sex and Gender in Research

Laboratory animals

We crossed the layer 5 driver mouse expressing Cre recombinase Tg(Rbp4-cre)KL100Gsat/Mmucd (Rbp4-Cre, The Jackson Laboratory, MMRRC:031125-UCD) with the inhibitory DREADD line B6.129-Gt(ROSA)26Sortm1(CAG-CHRM4*,-mCitrine)Ute/J (The Jackson Laboratory, No: 026219) to generate recombinant 'inhibitory DREADD' mice abbreviated as Rbp4Cre;hM4Di and with the excitatory DREADD line B6N;129-Tg(CAG-CHR3*, mCitrine)1Ute/J (The Jackson Laboratory, No: 026220) to generate recombinant 'excitatory DREADD' mice abbreviated as Rbp4Cre;hM3Dq. In the inhibitory DREADD line, previously, the excitatory DREADD receptor was designed as a targeted insertion into the Gt(ROSA)26Sor locus. However, in 2017, The Jackson Laboratory reported and confirmed a randomly integrated construct CAG-LSL-hM3Dq-pta-mCitrine instead of Gt(ROSA)26Sor locus and that the random insertion has no effect on the functionality of the allele. All animals were held in individually ventilated cages (IVCs) on a 12-hour light/dark cycle in the Biomedical Sciences Building (BSB), Oxford. Water and food were given ad libitum.

Wild animals

n/a

Reporting on sex

Inhibitory DREADD:
- mouse ID (A) male + 5mg/kg CNO;
- mouse ID (B) male + 1mg/kg CNO;
- mouse ID (C) male + 0.9% Saline;
- mouse ID (D) female + 5mg/kg CNO;
- mouse ID (E) female + 1mg/kg CNO;
- mouse ID (F) female + 0.9% Saline;

Excitatory DREAD:
- mouse ID (2.2A) male + 10mg/kg CNO;
- mouse ID (2.2B) male + 0.9% Saline;
- mouse ID (2.2C) male + 10mg/kg CNO;
- mouse ID (2.2D) male + 0.9% Saline;
- mouse ID (2.2E) female + 0.9% Saline;
- mouse ID (2.2F) female + 10mg/kg CNO;
- mouse ID (2.2G) female + 10mg/kg CNO;
- mouse ID (2.2H) female + 0.9% Saline;
- mouse ID (3.2B) male + 0.5mg/kg CNO;
- mouse ID (3.2D) male + 0.1mg/kg CNO;
- mouse ID (3.2E) male + 0.9% Saline;
- mouse ID (3.1A) male + 1mg/kg CNO;
- mouse ID (3.1C) male + 0.05mg/kg CNO;
- mouse ID (3.1D) male + 0.05mg/kg CNO;
- mouse ID (3.1E) female + 0.1mg/kg CNO;
- mouse ID (3.1F) female + 0.9% Saline;

Control experiments:
Rbp4-Cre (cortical layer 5):
- mouse ID (12.2C) male + 0.9% Saline;
- mouse ID (12.2f) female + 0.9% Saline;
- mouse ID (43.1A) male + 10mg/kg CNO;
- mouse ID (43.1B) male + 10mg/kg CNO;
- mouse ID (43.1C) female + 10mg/kg CNO;
- mouse ID (43.1D) male + 0.9% Saline;
- mouse ID (43.1E) male + 0.9% Saline;
- mouse ID (43.1F) male + 10mg/kg CNO;
- mouse ID (43.1G) female + 10mg/kg CNO.

hM4Di (Inhibitory receptor): mouse ID (11.1A) male + 0.9% Saline; mouse ID (11.1B) male + 0.9% Saline;
- hM3Dq (Excitatory receptor): mouse ID (12.1e) female + 0.9% Saline; mouse ID (12.1f) female + 0.9% Saline;

Field-collected samples	n/a
Ethics oversight	All animal experiments were approved by a local ethical review committee and conducted in accordance with the UK Animals (Scientific Procedures) Act, 1986 (ASPA), under valid personal and project licences.

Note that full information on the approval of the study protocol must also be provided in the manuscript.
